# Seasonal variation in the bacterial microbiome of questing nymphal ticks in Missouri, United States

**DOI:** 10.3389/fmicb.2026.1863755

**Published:** 2026-07-03

**Authors:** Aidan J. DeSalle, Victor I. Agbajelola, Aaron C. Ericsson, Chi-Ren Shyu, Kannappan Palaniappan, Enbal Shacham, Ram K. Raghavan

**Affiliations:** 1 Department of Pathobiology and Integrative Biomedical Sciences, College of Veterinary Medicine, University of Missouri, Columbia, MO, United States; 2 MU Metagenomics Center (MUMC), College of Veterinary Medicine, University of Missouri, Columbia, MO, United States; 3 MU Institute for Data Science and Informatics, University of Missouri, Columbia, MO, United States; 4 Department of Electrical Engineering and Computer Science, University of Missouri, Columbia, MO, United States; 5 College for Public Health and Social Justice, Saint Louis University, St. Louis, MO, United States; 6 Department of Public Health, College of Health Sciences, University of Missouri, Columbia, MO, United States

**Keywords:** microbiome, Missouri, seasonal variation, tick-borne diseases, ticks

## Abstract

**Background:**

Seasonal environmental variation may influence the composition of tick-associated bacterial communities. This study assessed seasonal differences in the microbiome of questing nymphal ticks collected from Missouri, United States.

**Methods:**

Questing ticks were collected during early and late seasonal periods at a livestock-associated site in central Missouri. To minimize confounding by developmental stage, microbiome analyses were restricted to nymphal ticks. Bacterial communities were characterized using 16S rRNA gene sequencing. Alpha diversity (richness, Shannon, and Simpson indices), beta diversity (Jaccard and Bray–Curtis dissimilarities), and differential abundance analyses were performed. Community differences were evaluated using permutational multivariate analysis of variance (PERMANOVA).

**Results:**

Sequencing generated 984–101,293 reads per sample. Sequencing depth was strongly correlated with observed richness (*R*^2^ = 0.808, *p* = 2 × 10^−7^). Comparisons of non-rarefied and rarefied datasets revealed no significant differences between early- and late-season nymphal ticks in observed richness, Shannon diversity, or Simpson diversity (all *p* > 0.05). In contrast, beta-diversity analyses identified significant differences in bacterial community membership between seasonal groups based on Jaccard dissimilarity (PERMANOVA: *F* = 1.5, *R*^2^ = 0.066, *p* = 0.0102), whereas Bray–Curtis dissimilarity showed a non-significant trend toward seasonal separation (*F* = 2.2, *R*^2^ = 0.090, *p* = 0.0834). Differential abundance analysis identified 18 amplicon sequence variants (ASVs) with raw *p*-values < 0.05, of which one Rickettsia-associated ASV remained significant following false discovery rate correction.

**Conclusion:**

Seasonal differences in bacterial community composition were detected among nymphal ticks despite similar levels of microbial richness and alpha diversity. The enrichment of a Rickettsia-associated ASV in early-season ticks suggests that season may influence the occurrence of specific bacterial taxa within tick microbiomes. Further studies using higher-resolution sequencing and pathogen-specific approaches are needed to clarify the ecological significance of these seasonal patterns.

## 1 Introduction

Tick-borne diseases have increased substantially across the United States over the past several decades, representing a growing concern for public health, veterinary medicine, and wildlife management (Rosenberg et al., 2018; Eisen et al., 2017). Expanding tick distributions, prolonged seasonal activity, and increasing reports of tick-associated pathogens have been linked, in part, to climatic and environmental change (Ogden et al., 2006; Sonenshine, 2018). Variations in temperature, humidity, and seasonal duration can influence tick phenology, host-seeking behavior, survival, and geographic range expansion, thereby affecting the ecology of tick populations and the microorganisms they harbor (Estrada-Peña et al., 2012; Eisen et al., 2016; Agbajelola et al., 2025).

Ticks are obligate hematophagous ectoparasites that undergo hemimetabolous development through four life stages: egg, larva, nymph, and adult (Sonenshine and Roe, 2014). Completion of each developmental stage requires a blood meal from a vertebrate host, creating opportunities for the acquisition and maintenance of diverse microbial communities (de la Fuente et al., 2017). Historically, tick activity resumes following diapause as environmental conditions become favorable in spring. However, changing climatic conditions have been associated with earlier seasonal emergence, prolonged host-seeking activity, and extended periods of environmental exposure (Ogden et al., 2006; Sonenshine, 2018). These changes may alter the ecological interactions between ticks, hosts, and environmental microbial reservoirs.

In addition to serving as vectors of infectious agents, ticks harbor complex microbial communities composed of symbionts, commensals, and environmental microorganisms. The tick microbiome has been implicated in several aspects of tick biology, including development, nutrition, immunity, and vector competence (Narasimhan and Fikrig, 2015; Bonnet et al., 2017). Resident microbial communities may influence the establishment and persistence of other microorganisms within ticks through competitive or facilitative interactions (Narasimhan et al., 2014; Abraham et al., 2017). Consequently, understanding factors that shape microbiome composition is important for advancing knowledge of tick ecology and tick-associated microbial dynamics.

Environmental conditions are recognized as important determinants of microbial community structure across diverse ecosystems, yet relatively little is known about how seasonal variation influences the microbiome of questing ticks in the Midwestern United States. Previous studies have demonstrated that tick-associated microbial communities can vary according to geographic location, host associations, sex, and developmental stage (Brinkerhoff et al., 2020; Briggs et al., 2025). However, the extent to which bacterial community composition varies across seasonal periods within naturally occurring tick populations remains incompletely understood.

To address this knowledge gap, we investigated seasonal variation in the bacterial microbiome of questing ticks collected from a livestock-associated landscape in central Missouri. Specifically, we evaluated whether bacterial diversity, community composition, and taxonomic structure differed between early- and late-season collections. We hypothesized that bacterial community composition and the relative abundance of specific taxa would vary between seasonal periods. Improved understanding of seasonal microbiome dynamics may provide insights into the ecological factors associated with temporal variation in tick-associated microbial communities in the Midwestern United States.

## 2 Methods

### 2.1 Study setting

Tick samples were collected from South Farm (38.53571, −92.1532) in Columbia, Missouri, a livestock research facility owned and managed by the University of Missouri in Boone County. The study site consisted of a cattle pasture characterized by dense and diverse vegetation, heterogeneous terrain, and a small creek running through the property, providing suitable habitat for tick populations.

Sampling was conducted throughout the 2024 tick activity season, spanning early June through mid-November. For ecological comparisons, collections were grouped into two biologically relevant seasonal periods based on observed tick activity and life-stage distribution: early season (June), corresponding primarily to peak nymphal and adult questing activity, and late season (July–November), characterized by continued nymphal activity and larvae emergence. This grouping was designed to reflect seasonal shifts in tick phenology rather than equivalent calendar durations. Environmental conditions during the sampling period included temperatures ranging from 32 °F to 97 °F, with cumulative rainfall of approximately 4.36 inches.

### 2.2 Tick collection

Questing ticks were collected using standardized dragging and flagging techniques with a 1 m^2^ white flannel cloth. Sampling was conducted at least once per week, with one to two collectors working simultaneously. Collections were performed at different times of the day to account for diurnal variation in tick activity.

Sampling followed a fixed transect along the fence line of the pasture, consisting of approximately 75 sampling points spaced ~10 m apart. A drag cloth attached to a 3-foot spindle rod and weighted with washers was pulled along the vegetation using a paracord. At each stop, ticks attached to the cloth were carefully removed and transferred into labeled collection tubes.

Each collection tube contained filter paper and plaster to maintain humidity and was sealed with a breathable latex cover. Metadata, including sampling time (start and end) and geographic location, were recorded for each collection event.

Following field collection, samples were placed in a portable cooler and transported to the One Health Laboratory in the Department of Pathobiology and Integrative Biomedical Sciences (PIBS), University of Missouri. Upon arrival, ticks were stored at −20 °C until further processing and identification.

### 2.3 Tick identification and classification

Frozen tick samples were thawed and examined under a stereomicroscope for morphological identification. Specimens were identified to genus, sex, and developmental stage using a standard dichotomous key (United States Department of Agriculture, Animal and Plant Health Inspection Service, 1976; Walker et al., 2003). Following identification, ticks were categorized based on species, life stage, and sex and assigned to labeled tubes accordingly. Prior to DNA extraction, ticks were surface sterilized by sequential washing in phosphate-buffered saline (PBS), 70% ethanol, and a final PBS rinse to minimize external contamination. Ticks were subsequently pooled into groups of 3–5 individuals sharing the same species and life stage, pooling was therefore structured by species and life stage rather than performed randomly. Each pooled tube was treated as an independent biological replicate for sequencing and downstream statistical analyses.

### 2.4 DNA extraction

Subsequently, ticks were transported to the University of Missouri Metagenomics Center (MUMC), and genomic DNA was extracted using the QIAamp PowerFecal Pro DNA extraction kit (Qiagen), following the manufacturer’s protocol with a modification: samples were homogenized in bead tubes using a TissueLyser II (Qiagen, Hilden, Germany) for 10 min at 30 Hz, instead of using the vortex adapter described in the protocol. DNA was eluted in 100 μL of elution buffer (Qiagen). DNA yields were quantified via fluorometry (Qubit 2.0, Invitrogen, Carlsbad, CA) using quant-iT BR dsDNA reagent kits (Invitrogen) and normalized to a uniform concentration and volume.

### 2.5 16S rRNA amplicon PCR and library preparation

Library preparation and sequencing were conducted at the University of Missouri (MU) Genomics Technology Core. Bacterial 16S rRNA amplicons were generated by amplifying the V4 region of the 16S rRNA gene using universal primers (U515F/806R) flanked by Illumina standard adapter sequences (Walters et al., 2011; Caporaso et al., 2011). Dual-indexed forward and reverse primers were used for all reactions.

PCR reactions (50 μL) contained 100 ng metagenomic DNA, 0.2 μM of each primer, 200 μM dNTPs, and 1 U of Phusion High-Fidelity DNA Polymerase (Thermo Fisher). The amplification conditions were as follows: initial denaturation at 98 °C for 3 min, followed by 25 cycles of denaturation (98 °C for 15 s), annealing (50 °C for 30 s), and extension (72 °C for 30 s), with a final extension at 72 °C for 7 min.

Amplicon pools (5 μL per reaction) were combined, thoroughly mixed, and purified using Axygen Axyprep MagPCR clean-up beads. Beads were added in a 1:1 ratio (50 μL beads per 50 μL amplicon pool) and incubated at room temperature for 15 min. The purified products were washed multiple times with 80% ethanol, dried, and resuspended in 32.5 μL of EB buffer (Qiagen). Samples were incubated at room temperature for 2 min, then on a magnetic stand for 5 min.

### 2.6 Sequencing

The final amplicon pool was evaluated using the Advanced Analytical Fragment Analyzer automated electrophoresis system, quantified with quantiTHS dsDNA reagent kits, and diluted according to Illumina’s standard protocol. Sequencing was performed on the Illumina MiSeq platform, generating 2 × 250 bp paired-end reads.

### 2.7 Informatics analysis

DNA sequences were processed at the MU Bioinformatics and Analytics Core. Primer sequences were removed from the 5′ ends of forward and reverse reads using Cutadapt version 2.6 (Martin, 2011). Reads lacking a detectable 5′ primer were discarded, with an allowable error rate of 0.1 and a minimum overlap of 3 bp with the primer sequence required for trimming.

Denoising, dereplication, chimera removal, and amplicon sequence variant (ASV) inference were performed using the DADA2 plugin implemented in QIIME2 version 1.10.0 (Callahan et al., 2016). Forward and reverse reads were truncated to 150 bp, reads with more than two expected errors were discarded, and chimeric sequences were identified and removed using the consensus method. Taxonomic assignments were generated using the classify-sklearn algorithm in QIIME2 against the SILVA v132 reference database (Pruesse et al., 2007).

Differential abundance analysis was conducted at the ASV level using Mann–Whitney rank-sum tests to compare normalized ASV abundances between early- and late-season nymphal tick pools (Mann and Whitney, 1947). To account for multiple hypothesis testing, *p*-values were adjusted using the Benjamini–Hochberg false discovery rate (FDR) procedure (Benjamini and Hochberg, 1995). ASVs with raw *p*-values < 0.05 were considered differentially abundant for exploratory analyses, whereas ASVs with FDR-adjusted *p*-values < 0.05 were considered statistically significant. This approach has been widely applied in microbiome studies to identify differentially abundant taxa while controlling for false-positive discoveries (Weiss et al., 2017; Nearing et al., 2022).

### 2.8 Statistical analysis

All downstream analyses were restricted to nymphal tick pools to minimizcontrollingfounding effects of developmental stage on microbiome composition. Alpha-diversity metrics, including observed ASV richness, Shannon diversity, and Simpson diversity, were calculated using both non-rarefied and rarefied datasets. Because sequencing depth was significantly correlated with observed richness, samples were rarefied to 1,165 reads per sample prior to final alpha-diversity comparisons. Differences in sequencing depth, richness, and diversity indices between early- and late-season groups were evaluated using Mann–Whitney rank-sum tests implemented in PAST version 4.03 (Hammer et al., 2001), with statistical significance defined as *p* < 0.05.

Beta-diversity analyses were conducted using Jaccard and Bray–Curtis dissimilarity metrics to assess differences in bacterial community composition between seasonal groups. Principal coordinate analysis (PCoA) was used to visualize community clustering patterns. Permutational multivariate analysis of variance (PERMANOVA) was performed in PAST version 4.03 using 9,999 permutations to evaluate differences in community composition between early- and late-season nymphal tick pools. Effect sizes were reported as *R*^2^ values.

To assess the assumption of homogeneity of multivariate dispersion, permutational analysis of multivariate dispersions (PERMDISP) was performed using MicrobiomeAnalyst (Lu et al., 2023). Differentially abundant ASVs were visualized using volcano plots and heatmaps generated from normalized abundance data. Statistical significance was assessed at *p* < 0.05 unless otherwise stated.

## 3 Results

### 3.1 Tick collections and nymphal pools included in microbiome analyses

A total of 1,625 ticks representing four species and multiple life stages were collected across the early- and late-season sampling periods (Table 1). Early-season collections consisted exclusively of nymphal and adult ticks, with no larvae detected. *Amblyomma americanum* was the predominant species during this period, accounting for 70 nymphs and 31 adults. Other species were collected in substantially lower numbers, including *Haemaphysalis longicornis* (2 nymphs and 1 adult), *Haemaphysalis leporispalustris* (1 nymph), and *Dermacentor* var*iabilis* (3 adults). Overall, 73 nymphs and 35 adults were collected during the early season.

**Table 1:** Species composition, life-stage distribution, and nymphal pools included in microbiome analyses across early- and late-season collections.

Species	Early season larvae	Early season nymphs	Early season adults	Early season nymph pools	Late season larvae	Late season nymphs	Late season adults	Late season nymph pools
*A. americanum*	0	70	31	9	1,424	78	4	14
*H. longicornis*	0	2	1	1	0	0	5	0
*H. leporispalustris*	0	1	0	1	0	0	0	0
*D. variabilis*	0	0	3	0	6	0	0	0
Total	0	73	35	11	1,430	78	9	14

Ticks were pooled prior to DNA extraction. Nymphal pools contained 3–6 ticks per pool and were treated as independent biological replicates for sequencing and downstream statistical analyses. Following revision of the study design, microbiome analyses were restricted to nymphal pools (11 early-season pools and 14 late-season pools; 25 pools total). Late-season nymphal pools consisted exclusively of *Amblyomma americanum*, whereas early-season pools included separate pools of *A. americanum*, *Haemaphysalis longicornis*, and *Haemaphysalis leporispalustris*.

In contrast, late-season collections were dominated by larvae. A total of 1,430 larvae were collected, of which 1,424 were identified as *A. americanum*. This species also accounted for all 78 nymphs and 4 adults collected during the late season. Additional species identified during this period included *H. longicornis* (5 adults) and *D. variabilis* (6 larvae), while *H. leporispalustris* was not detected. Overall, late-season collections comprised 1,430 larvae, 78 nymphs, and 9 adults.

Because substantial differences in life-stage composition were observed between seasonal collections, microbiome analyses were restricted to nymphal ticks to minimize potential confounding effects of developmental stage. Of the 41 pools initially prepared, 25 nymphal pools were retained for the final analysis, comprising 11 early-season pools and 14 late-season pools. These pools represented 151 nymphs in total. Late-season pools consisted exclusively of *A. americanum* nymphs, whereas early-season pools included separate pools of *A. americanum*, *H. longicornis*, and *H. leporispalustris* nymphs to ensure species-specific representation.

### 3.2 Alpha diversity of the nymphal tick microbiome

#### 3.2.1 Sequencing depth and alpha diversity based on non-rarefied data

**Figure 1:**
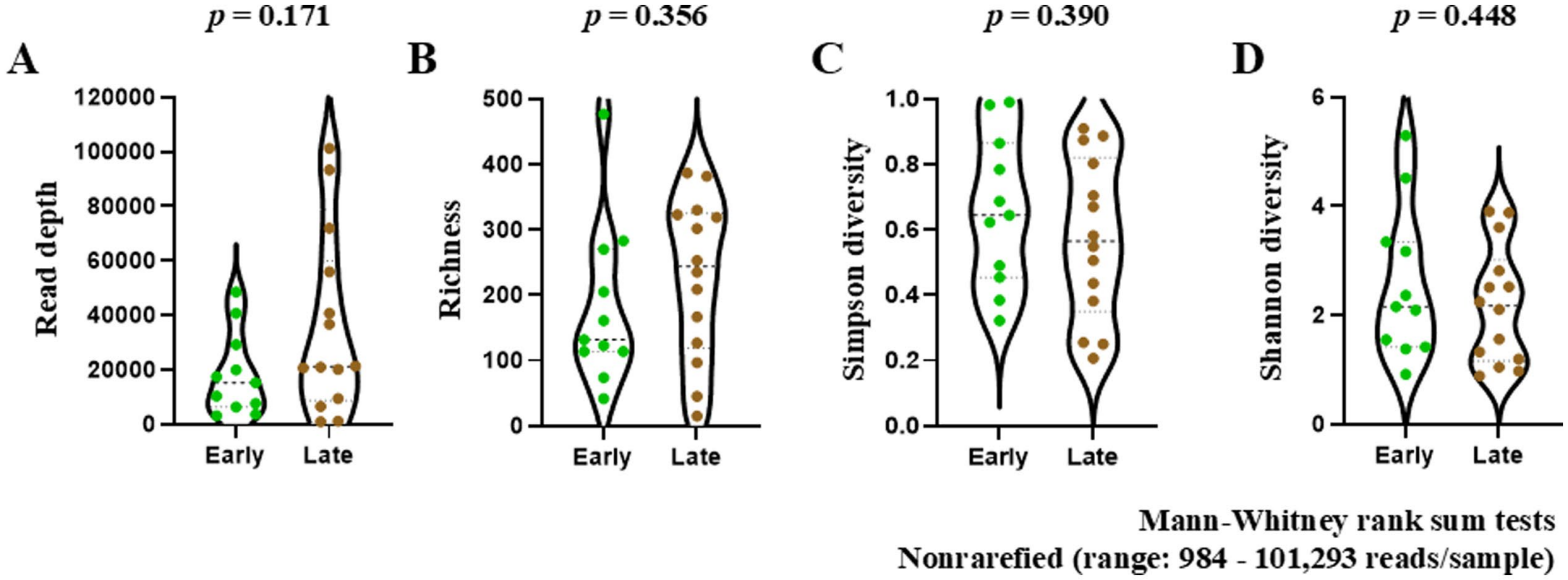
Sequencing depth and alpha diversity comparisons between early- and late-season ticks using non-rarefied data. Comparison of sequencing depth and alpha-diversity metrics between early- and late-season nymphal tick pools using non-rarefied 16S rRNA gene sequence data. **(A)** Sequencing depth (reads per sample), **(B)** observed richness (number of ASVs), **(C)** Simpson diversity index, and **(D)** Shannon diversity index. Each point represents an individual pooled sample, and violin plots illustrate the distribution of values within each seasonal group. Statistical comparisons were performed using Mann–Whitney rank-sum tests. No significant differences were detected between seasonal groups for any metric (*p* > 0.05).

High-throughput sequencing of bacterial 16S rRNA gene amplicons generated between 984 and 101,293 reads per sample. Comparison of non-rarefied sequence data revealed no significant difference in sequencing depth between early- and late-season nymphal tick pools (Mann–Whitney rank-sum test, *p* = 0.171; Figure 1A). Similarly, microbial richness, measured as the number of observed amplicon sequence variants (ASVs), did not differ significantly between seasonal groups (*p* = 0.356; Figure 1B).

Measures of community diversity also showed no significant seasonal variation. Simpson diversity was comparable between early- and late-season nymphal pools (*p* = 0.390; Figure 1C), and no significant difference was observed for Shannon diversity (*p* = 0.448; Figure 1D). Collectively, these findings indicate that the richness and within-sample diversity of bacterial communities were similar between early- and late-season nymphs when assessed using non-rarefied sequence data.

#### 3.2.2 Relationship between sequencing depth and microbial richness

**Figure 2:**
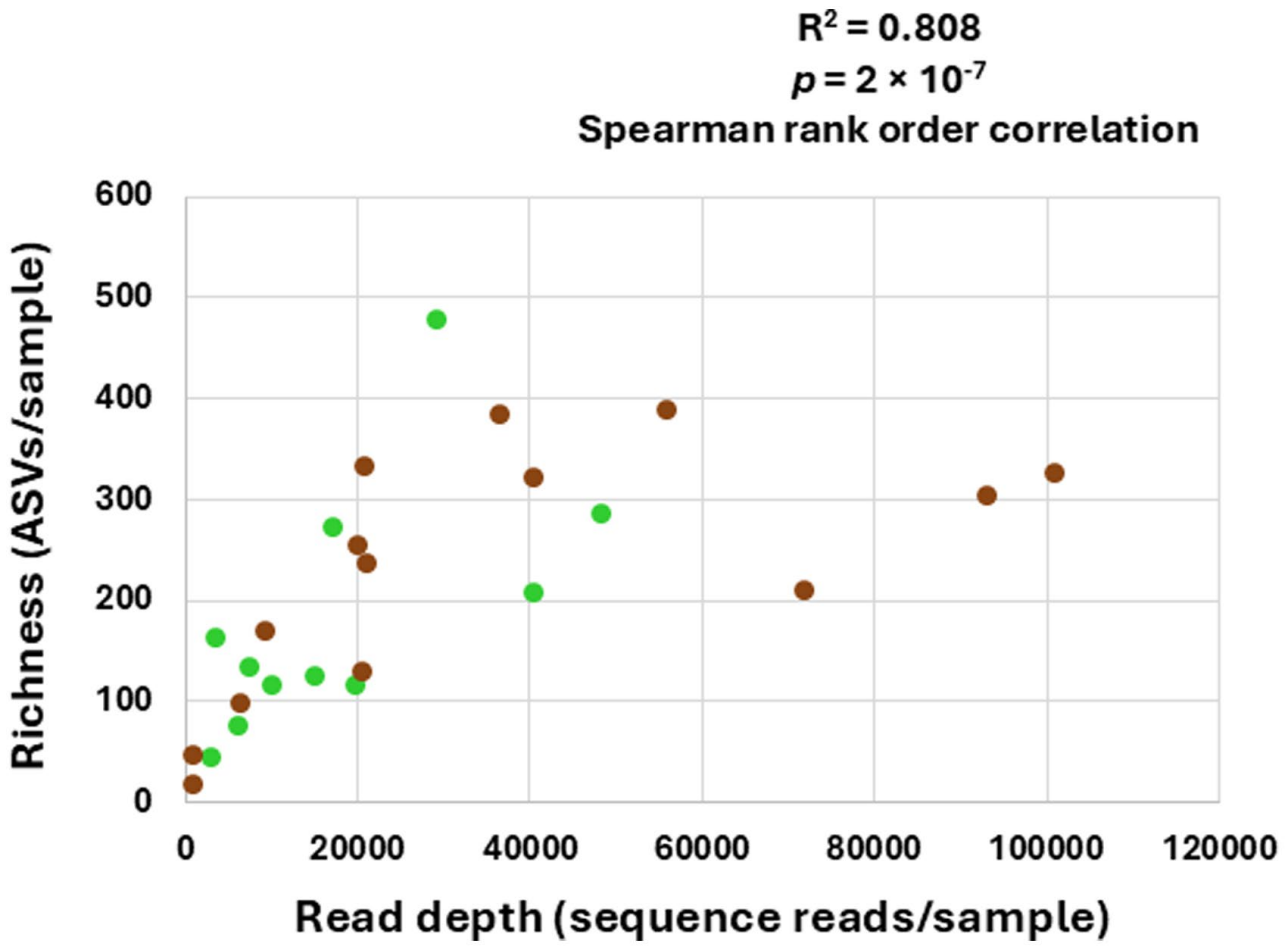
Relationship between sequencing depth and observed microbial richness across nymphal tick pools. Scatter plot illustrating the relationship between sequencing depth (number of reads per sample) and microbial richness (number of observed ASVs). A strong positive correlation was observed between sequencing depth and richness (Spearman rank correlation, *R*^2^ = 0.808, *p* = 2 × 10^−7^), indicating that samples with greater sequencing depth tended to have higher detected ASV richness. This relationship supported rarefaction prior to downstream alpha-diversity analyses.

Because sequencing depth varied among nymphal tick pools, the relationship between read depth and observed ASV richness was examined. A strong positive correlation was detected between sequencing depth and richness (Spearman rank correlation, *R*^2^ = 0.808, *p* = 2 × 10^−7^; Figure 2). Samples with greater sequencing depth yielded higher numbers of detected ASVs, indicating that sequencing effort influenced richness estimates. This finding supported rarefaction of the dataset prior to subsequent alpha-diversity comparisons.

#### 3.2.3 Alpha diversity following rarefaction

**Figure 3:**
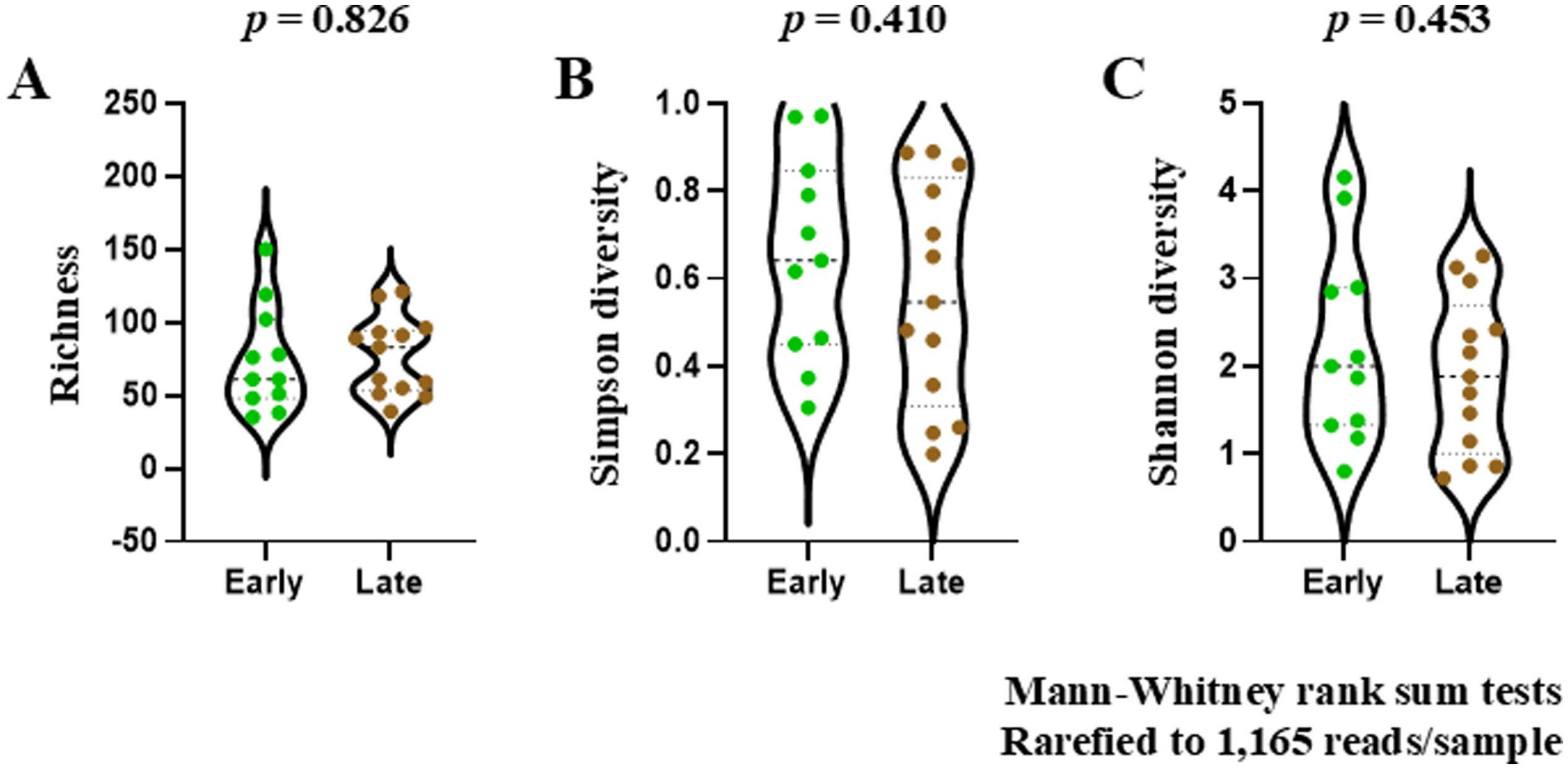
Alpha diversity metrics following rarefaction of sequencing depth. Violin plots show comparisons between early- and late-season ticks after rarefying samples to an equal sequencing depth. **(A)** Observed richness (number of ASVs), **(B)** Simpson diversity index, and **(C)** Shannon diversity index. Each point represents an individual pooled sample, and violin plots illustrate the distribution of values within each seasonal group. Statistical comparisons were performed using Mann–Whitney rank-sum tests. No significant differences were observed between seasonal groups for richness, Simpson diversity, or Shannon diversity (*p* > 0.05).

To account for variation in sequencing depth among samples, sequence data were rarefied to 1,165 reads per sample and alpha-diversity analyses were repeated (Figure 3). Following rarefaction, microbial richness remained similar between early- and late-season nymphal pools (*p* = 0.826; Figure 3A). Likewise, no significant differences were detected in Simpson diversity (*p* = 0.410; Figure 3B) or Shannon diversity (*p* = 0.453; Figure 3C). The consistency of results before and after rarefaction indicates that the absence of seasonal differences in richness and alpha diversity was not attributable to unequal sequencing depth. Overall, these findings suggest that early- and late-season nymphal ticks harbor bacterial communities with comparable levels of taxonomic richness and within-sample diversity.

### 3.3 Seasonal differences in microbial community composition (beta diversity)

**Figure 4:**
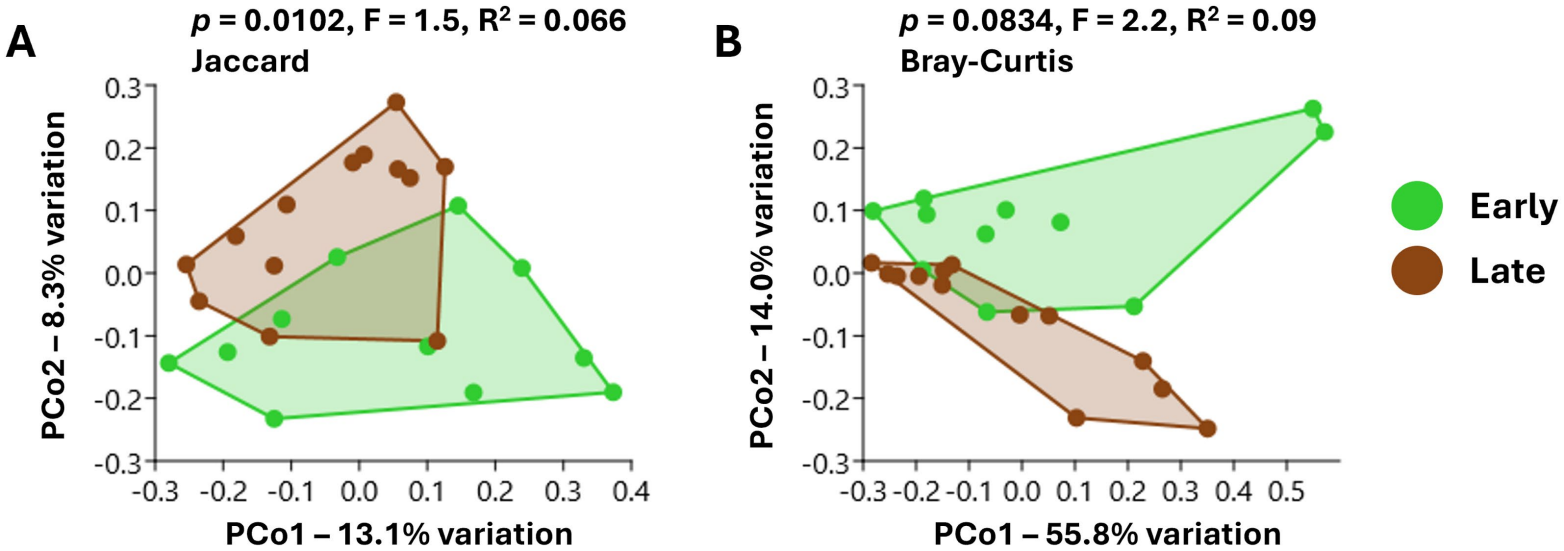
Seasonal differences in beta diversity among nymphal tick microbiomes. Principal coordinate analysis (PCoA) based on **(A)** Jaccard dissimilarity and **(B)** Bray–Curtis dissimilarity comparing bacterial communities in early- and late-season nymphal tick pools. Each point represents an individual pooled sample, with colors indicating seasonal group. Statistical differences in community composition were evaluated using permutational multivariate analysis of variance (PERMANOVA). Jaccard dissimilarity revealed significant differences in community membership between seasons (*F* = 1.5, *R*^2^ = 0.066, *p* = 0.0102), whereas Bray–Curtis dissimilarity showed a non-significant trend toward seasonal separation (*F* = 2.2, *R*^2^ = 0.090, *p* = 0.0834).

Although alpha-diversity metrics did not differ significantly between early- and late-season nymphal tick pools, analyses of beta diversity revealed evidence of seasonal variation in overall microbial community composition (Figure 4).

Using Jaccard dissimilarity, which evaluates differences in community membership based on the presence or absence of taxa, microbial communities differed significantly between seasonal groups (PERMANOVA: *F* = 1.5, *R*^2^ = 0.066, *p* = 0.0102; Figure 4A). Ordination analysis demonstrated clustering of early- and late-season samples, indicating that season accounted for approximately 6.6% of the variation in bacterial community composition and influenced the occurrence of bacterial taxa within nymphal ticks.

In contrast, Bray–Curtis dissimilarity, which incorporates relative taxon abundance, revealed a trend toward seasonal differentiation that did not reach statistical significance (PERMANOVA: *F* = 2.2, *R*^2^ = 0.090, *p* = 0.0834; Figure 4B). Nevertheless, visual inspection of the ordination plot suggested partial separation between seasonal groups, indicating that season explained approximately 9.0% of the variation in abundance-weighted community composition.

Collectively, these findings suggest that seasonal variation was associated primarily with differences in bacterial community membership rather than large shifts in the relative abundance of dominant taxa. While the presence or absence of bacterial taxa differed significantly between seasonal groups, abundance-based differences were comparatively weaker and did not remain statistically significant.

### 3.4 Differential abundance analysis identifies seasonally enriched taxa

**Figure 5:**
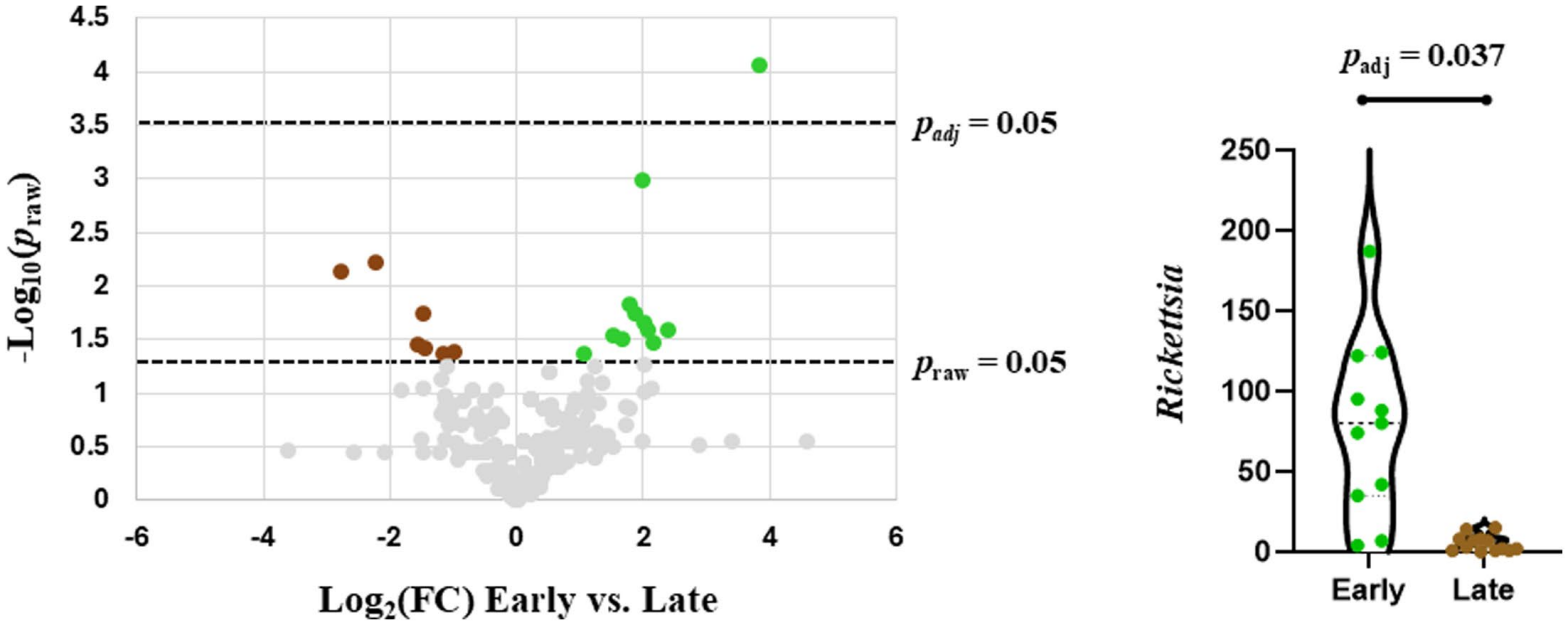
Differential abundance analysis of bacterial ASVs between early- and late-season nymphal tick pools. The volcano plot displays log₂ fold changes in ASV abundance between seasonal groups plotted against statistical significance (−log₁₀ *p* value). Colored points represent ASVs with raw *p* values < 0.05. Horizontal dashed lines indicate thresholds for raw (*p* = 0.05) and false discovery rate (FDR)-adjusted significance (*p* = 0.05). Eighteen ASVs exhibited raw *p* values < 0.05, but only one ASV remained significant after FDR correction. The violin plot illustrates normalized read counts of the Rickettsia ASV, which was significantly enriched in early-season nymphal ticks (adjusted *p* = 0.037).

To identify specific bacterial taxa associated with seasonal differences in microbial community composition, differential abundance analyses were performed at the amplicon sequence variant (ASV) level. A total of 18 ASVs exhibited significant differences between early- and late-season nymphal tick pools based on raw *p* values (*p* < 0.05) (Figure 5). Following false discovery rate (FDR) correction for multiple testing, only a single ASV remained statistically significant. This ASV was taxonomically classified as *Rickettsia* and was significantly enriched in early-season nymphal ticks (adjusted *p* = 0.037; Figure 5).

The volcano plot further demonstrated that most differentially abundant ASVs exhibited positive log₂ fold-change values, indicating higher abundance in early-season samples relative to late-season samples. Examination of normalized read counts revealed substantially greater abundance of the *Rickettsia* ASV in early-season nymphs, whereas late-season samples contained few or no reads corresponding to this taxon (Figure 5). This pattern suggests a pronounced seasonal association of *Rickettsia* within the nymphal tick microbiome.

### 3.5 Hierarchical clustering of differentially abundant ASVs

**Figure 6:**
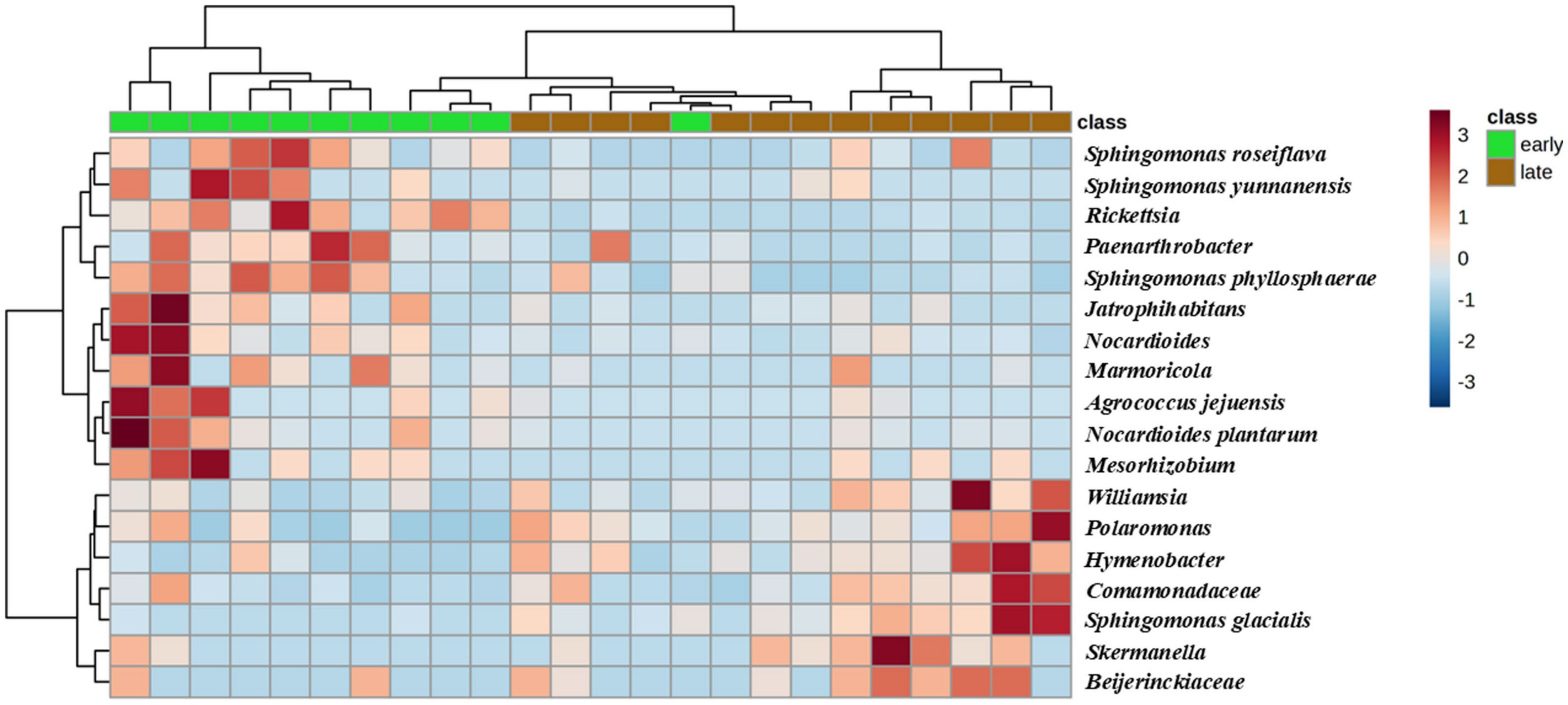
Hierarchical clustering heatmap of seasonally associated bacterial taxa. Heatmap showing the relative abundance of the 18 ASVs exhibiting raw *p* values < 0.05 in differential abundance testing. Columns represent individual nymphal tick pools and rows represent ASVs. Samples were clustered using the unweighted pair group method with arithmetic mean (UPGMA), and heatmap colors represent standardized abundance values (z-scores). Green and brown bars above the heatmap denote early- and late-season samples, respectively. Clustering revealed substantial grouping of samples by season and highlighted bacterial taxa exhibiting consistent seasonal enrichment patterns.

To further investigate seasonal patterns in bacterial community composition, the relative abundances of the 18 ASVs identified through differential abundance testing (*p* < 0.05) were visualized using hierarchical clustering and heatmap analysis (Figure 6). Samples clustered largely according to season, with early- and late-season nymphal pools forming distinct groups. Several bacterial taxa, including *Rickettsia*, *Nocardioides*, *Sphingomonas phyllosphaerae*, *Sphingomonas roseiflava*, *Sphingomonas yunnanensis*, *Paenarthrobacter*, *Marmoricola*, and *Agrococcus jejuensis*, exhibited higher relative abundance in early-season samples.

Conversely, taxa including *Williamsia*, *Polaromonas*, *Hymenobacter*, members of the family *Comamonadaceae*, *Sphingomonas glacialis*, *Skermanella*, and *Beijerinckiaceae* were more abundant in late-season samples. The clustering pattern indicates that seasonal differences in microbiome composition were driven by coordinated shifts in multiple bacterial taxa rather than by a single dominant organism. Although only the *Rickettsia* ASV remained significant after correction for multiple testing, the heatmap demonstrates that numerous environmentally associated bacterial taxa exhibited consistent seasonal trends across samples.

## 4 Discussion

This study evaluated seasonal variation in the bacterial microbiome of questing nymphal ticks collected from a livestock-associated environment in Missouri. Although microbial richness and alpha-diversity metrics did not differ significantly between early- and late-season collections, analyses of community composition revealed evidence of seasonal structuring of the tick microbiome. Significant differences in community membership were detected using Jaccard dissimilarity, and differential abundance analyses identified a *Rickettsia*-associated amplicon sequence variant (ASV) that was significantly enriched in early-season ticks after correction for multiple testing. Collectively, these findings indicate that seasonal environmental conditions may influence the occurrence of specific bacterial taxa within tick-associated microbial communities, even when overall bacterial diversity remains relatively stable.

The absence of significant seasonal differences in microbial richness, Shannon diversity, and Simpson diversity suggests that the overall diversity of bacterial communities within nymphal ticks remained relatively consistent throughout the sampling period. Similar observations have been reported in studies of tick microbiomes across different geographic regions, where alpha-diversity metrics were often less sensitive to environmental variation than measures of community composition (Van Treuren et al., 2015; Chicana et al., 2019; Xu et al., 2024). The strong correlation observed between sequencing depth and ASV richness further highlights the importance of accounting for sequencing effort when comparing microbial communities, a pattern that has been widely documented in microbial ecology studies (Callahan et al., 2016; Weiss et al., 2017). Following rarefaction, richness and diversity metrics remained non-significant, suggesting that seasonal effects were not reflected in overall diversity but rather in the composition of bacterial assemblages.

In contrast to alpha diversity, beta-diversity analyses revealed evidence of seasonal differences in microbial community composition. Jaccard dissimilarity demonstrated significant separation between early- and late-season samples, indicating that the presence or absence of bacterial taxa differed between seasonal groups. Bray–Curtis dissimilarity showed a similar but non-significant trend, suggesting that seasonal effects were more strongly associated with community membership than with large shifts in the abundance of dominant taxa. Similar patterns have been reported in studies demonstrating that environmental conditions can influence which bacterial taxa colonize ticks without necessarily altering overall community diversity (Van Treuren et al., 2015; Chicana et al., 2019; Tonk-Rügen et al., 2023).

Seasonal differences in tick-associated microbial communities may reflect variation in environmental and ecological conditions across the sampling period. Factors such as temperature, humidity, vegetation structure, and host availability have been identified as important drivers of microbial community assembly in previous studies (Brinkerhoff et al., 2020; Briggs et al., 2025), although these variables were not directly measured in the present investigation. In the central Midwestern United States, including Missouri, tick activity and host-seeking behavior are strongly shaped by seasonal environmental conditions (Bouzek et al., 2013; Raghavan et al., 2021; McClung et al., 2023). As ticks interact with changing environmental and host-associated microbial reservoirs throughout the year, they may acquire distinct bacterial assemblages that contribute to temporal variation in microbiome composition. Similar ecological mechanisms have been proposed to explain seasonal shifts in tick-associated microbiota in other studies (Brinkerhoff et al., 2020; Briggs et al., 2025).

While these findings suggest an association between season and bacterial community composition, other ecological factors may also have contributed to the observed patterns. Although *A. americanum* constituted the vast majority of nymphs included in the analysis, early-season collections also contained a small number of *H. longicornis* and *H. leporispalustris* nymphs, whereas late-season samples consisted exclusively of *A. americanum*. Because tick species can harbor distinct microbial communities (Van Treuren et al., 2015; Brinkerhoff et al., 2020), species-specific differences may have contributed to some of the observed variation in microbiome structure. In addition, the seasonal groupings were based on biologically relevant differences in tick phenology rather than equivalent calendar durations, with the late-season period encompassing a broader temporal window. Consequently, the microbiome patterns observed in this study likely reflect a combination of seasonal influences, species composition, and temporal heterogeneity within naturally occurring tick populations.

Differential abundance analyses further supported the presence of seasonal microbiome variation. Eighteen ASVs exhibited raw *p*-values below 0.05, although only one remained significant after correction for multiple testing. This ASV was classified within the genus Rickettsia and was enriched in early-season nymphal ticks. Members of the genus *Rickettsia* are frequently reported in tick microbiomes and include both vertically transmitted endosymbionts and medically important pathogens (Narasimhan and Fikrig, 2015; Bonnet et al., 2017; Brinkerhoff et al., 2020). Because 16S rRNA sequencing does not provide sufficient taxonomic resolution to distinguish among Rickettsia species, it is not possible to determine whether the detected ASV represents a pathogenic or non-pathogenic lineage. Nevertheless, the enrichment of a Rickettsia-associated ASV in early-season ticks suggests that seasonal factors may influence the occurrence of specific microbial taxa within tick populations.

Several additional taxa showing seasonal enrichment, including *Nocardioides*, *Sphingomonas phyllosphaerae*, and *Paenarthrobacter*, are commonly associated with soil and plant-associated environments (Leys et al., 2004; Busse, 2016). These taxa have been reported in environmental microbiomes and occasionally detected in arthropods, likely reflecting acquisition from vegetation and surrounding habitats during host-seeking activity. Their occurrence within tick microbiomes supports the concept that environmental reservoirs contribute to shaping microbial communities associated with questing ticks.

From a One Health perspective, understanding factors that influence the composition of tick-associated microbial communities is important because ticks serve as interfaces between wildlife, domestic animals, and humans. Although this study did not directly assess pathogen prevalence, microbial community structure may provide insights into ecological processes influencing tick-associated microorganisms. Continued characterization of seasonal microbiome dynamics may improve understanding of how environmental conditions shape microbial assemblages within ticks and may help inform future studies investigating tick-borne pathogen ecology.

### 4.1 Study limitations

Several limitations should be considered when interpreting the findings of this study. First, bacterial communities were characterized using 16S rRNA gene sequencing, which provides limited taxonomic resolution and does not allow definitive identification of bacterial species or discrimination between pathogenic and non-pathogenic strains. Consequently, functional interpretations regarding pathogen transmission, microbial interactions, or vector competence cannot be directly inferred from these data.

Second, ticks were pooled prior to DNA extraction to ensure sufficient DNA yield for sequencing. While pooling facilitated characterization of population-level microbial patterns, it may have obscured variation among individual ticks and reduced the detection of rare taxa. In addition, sampling was conducted at a single livestock-associated site in central Missouri, which may limit the generalizability of the findings to other geographic regions, habitats, or tick populations.

Third, although microbiome analyses were restricted to nymphal ticks to minimize confounding associated with developmental stage, species composition differed slightly between seasonal groups. Early-season samples included a small number of *H. longicornis* and *H. leporispalustris* nymphs in addition to *A. americanum*, whereas all late-season nymphs were *A. americanum*. Because tick species can harbor distinct microbial communities, some of the observed differences in community composition may reflect species-specific microbiome characteristics rather than seasonal variation alone.

Furthermore, seasonal groupings were based on biologically relevant differences in tick phenology rather than equivalent calendar durations, with the early season represented by June collections and the late season spanning July through November. Although this approach reflects natural patterns of tick activity, the unequal temporal windows may have contributed to variability in microbial community composition and should be considered when interpreting seasonal comparisons.

Finally, this study was observational in nature and therefore cannot establish causal relationships between seasonal environmental conditions and microbiome composition. Future studies incorporating longitudinal sampling, larger species-specific datasets, environmental measurements, and pathogen-specific molecular assays will be valuable for disentangling the relative contributions of season, host ecology, and tick species to microbiome structure and for clarifying the ecological significance of seasonal microbiome variation.

## 5 Conclusion

This study demonstrates that the bacterial microbiome of questing nymphal ticks in Missouri exhibits seasonal variation in community composition, despite relatively stable levels of microbial richness and alpha diversity. Significant differences in bacterial community membership were observed between early- and late-season collections, and a *Rickettsia*-associated ASV was significantly enriched in early-season ticks. These findings suggest that seasonal environmental conditions may influence the occurrence of specific bacterial taxa within tick microbiomes. Further investigations using higher-resolution sequencing approaches, pathogen-specific molecular assays, and longitudinal sampling designs are needed to clarify the ecological significance of these seasonal patterns and their potential relevance to tick-borne disease ecology.

## Data Availability

All 16S rRNA amplicon sequencing data supporting the current study are available at the National Center for Biotechnology Information (NCBI) Sequence Read Archive (SRA) as BioProject PRJNA1447488.
